# Characterization of esophageal motor activity, gastroesophageal reflux, and evaluation of prokinetic effectiveness in mechanically ventilated critically ill patients: a high-resolution impedance manometry study

**DOI:** 10.1186/s13054-021-03479-8

**Published:** 2021-02-08

**Authors:** Karel Balihar, Jan Kotyza, Lucie Zdrhova, Jana Kozeluhova, Michal Krcma, Martin Matejovic

**Affiliations:** Department of Internal Medicine, Faculty of Medicine in Pilsen, Pilsen University Hospital, Charles University Prague, Alej Svobody 80, 30406 Pilsen, Czech Republic

**Keywords:** Critical illness, Esophageal dysfunction, High-resolution impedance manometry, Prokinetics, Gastroesophageal reflux

## Abstract

**Background:**

Motility disorders of upper gastrointestinal tract are common in critical illness and associated with significant clinical consequences. However, detailed quantitative and qualitative analyses of esophageal motor functions are lacking. Therefore, we aimed to characterize the key features of esophageal motility functions using high-resolution impedance manometry (HRIM) and to evaluate an objective link between esophageal motor patterns, gastric emptying, and gastroesophageal reflux. We also studied the prokinetic effects of metoclopramide.

**Methods:**

We prospectively performed HRIM for 16 critically ill hemodynamically stable patients. Patients were included if they had low gastric volume (LGV; < 100 mL/24 h, *n* = 8) or high gastric volume (HGV; > 500 mL/24 h, *n* = 8). The HRIM data were collected for 5 h with intravenous metoclopramide administration (10 mg) after the first 2 h.

**Results:**

The findings were grossly abnormal for all critically ill patients. The esophageal contraction vigor was markedly increased, indicating prevailing hypercontractile esophagus. Ineffective propulsive force was observed for 73% of esophageal activities. Panesophageal pressurization was the most common pressurization pattern (64%). Gastroesophageal reflux predominantly occurred with transient lower esophageal sphincter relaxation. The common features of the LGV group were a hyperreactive pattern, esophagogastric outflow obstruction, and frequent reflux. Ineffective motility with reduced lower esophageal sphincter tone, and paradoxically fewer reflux episodes, was common in the HGV group. Metoclopramide administration reduced the number of esophageal activities but did not affect the number of reflux episodes in either group.

**Conclusion:**

All critically ill patients had major esophageal motility abnormalities, and motility patterns varied according to gastric emptying status. Well-preserved gastric emptying and maintained esophagogastric barrier functions did not eliminate reflux. Metoclopramide failed to reduce the number of reflux episodes regardless of gastric emptying status.

*Trial registration* ISRCTN, ISRCTN14399966. Registered 3.9.2020, retrospectively registered. https://www.isrctn.com/ISRCTN14399966.

## Introduction

Dysfunction of the upper gastrointestinal tract (UGIT) leads to deterioration in the patient’s nutritional status, bacterial colonization, and increased risks of reflux esophagitis, and aspiration [1]. Unfortunately, there is very little quantitative and qualitative data regarding esophageal motility functions in mechanically ventilated critically ill patients. Kölbel et al. performed a pilot study of 15 mechanically ventilated patients, which revealed that they had decreased propulsive esophageal motility that was further impaired by any kind of sedation [2]. Nind et al. also evaluated 15 mechanically ventilated patients and reported that gastroesophageal reflux was predominantly related to low or absent lower esophageal sphincter (LES) pressure, which often coexisted with cough or straining [3]. However, those studies evaluated esophageal motility using conventional manometry, where the sensors are spaced at 3–5-cm intervals.

Unlike conventional manometry, high-resolution impedance manometry (HRIM) measures esophageal pressures with 423 sensors distributed longitudinally and radially in the esophagus, along with esophageal impedance monitoring (18 sensors), thus allowing for a very detailed evaluations of esophageal pressures, peristalsis, sphincter functions, and reflux [4]. It might, therefore, identify relevant abnormalities not detected by conventional manometry. Among critically ill patients, pharyngeal HRIM has only recently been used in a study of post-extubation dysphagia [5]. Persson et al. used HRM to evaluate factors affecting esophageal pressures during changes in ventilator settings [6]. Ahlstrand et al. showed that muscle relaxation with rocuronium does not decrease the barrier pressure during anesthesia induction [7]. We believe that HRIM has great potential to improve our understanding of esophageal functions in critically ill patients. In particular, deeper insights into the complex relationships between esophageal motility patterns, antireflux barrier, and gastric emptying may pave way for more effective and safe treatment options and patient care. Therefore, the present mechanistic study used HRIM to characterize the features of esophageal motility in ventilated patients who were receiving intensive care, and evaluated the esophageal physiology and occurrence of reflux depending on the gastric nutritional tolerance. In addition, the effects of metoclopramide were evaluated and related to the esophageal motility patterns.

## Methods

### Study design, population, and setting

This prospective exploratory study evaluated adults (≥ 18 years) who were admitted to the medical intensive care unit of a tertiary academic referral center. The study was conducted in accordance with Good Clinical Practice, as defined by the International Conference on Harmonization, the ethical principles underlying European Union Directive 2001/20/EC, and all applicable local requirements. The study protocol was approved by the appropriate institutional review board and ethics committee. Informed consent was obtained from the patients’ next of kin, as well as the patients themselves once they had regained the capacity to provide informed consent.

Patients were considered eligible for the study if they were mechanically ventilated, hemodynamically stable (maximum stable dose of noradrenaline of 0.2 µg/kg/min), and had initiated gastric enteral nutrition. The patients were subclassified based on their residual gastric volume (GRV) at 24 h before enrolment as having either low residual gastric volume (LGV; ≤ 100 mL/24 h, *n* = 8) or high gastric volume (HGV; ≥ 500 mL/24 h, *n* = 8). Patients were excluded if they had GRV between 100 and 500 ml/24 h, a history of surgery for UGIT dysfunction, gastroesophageal reflux disease, portal hypertension, or active bleeding in the UGIT.

**Fig. 1:**
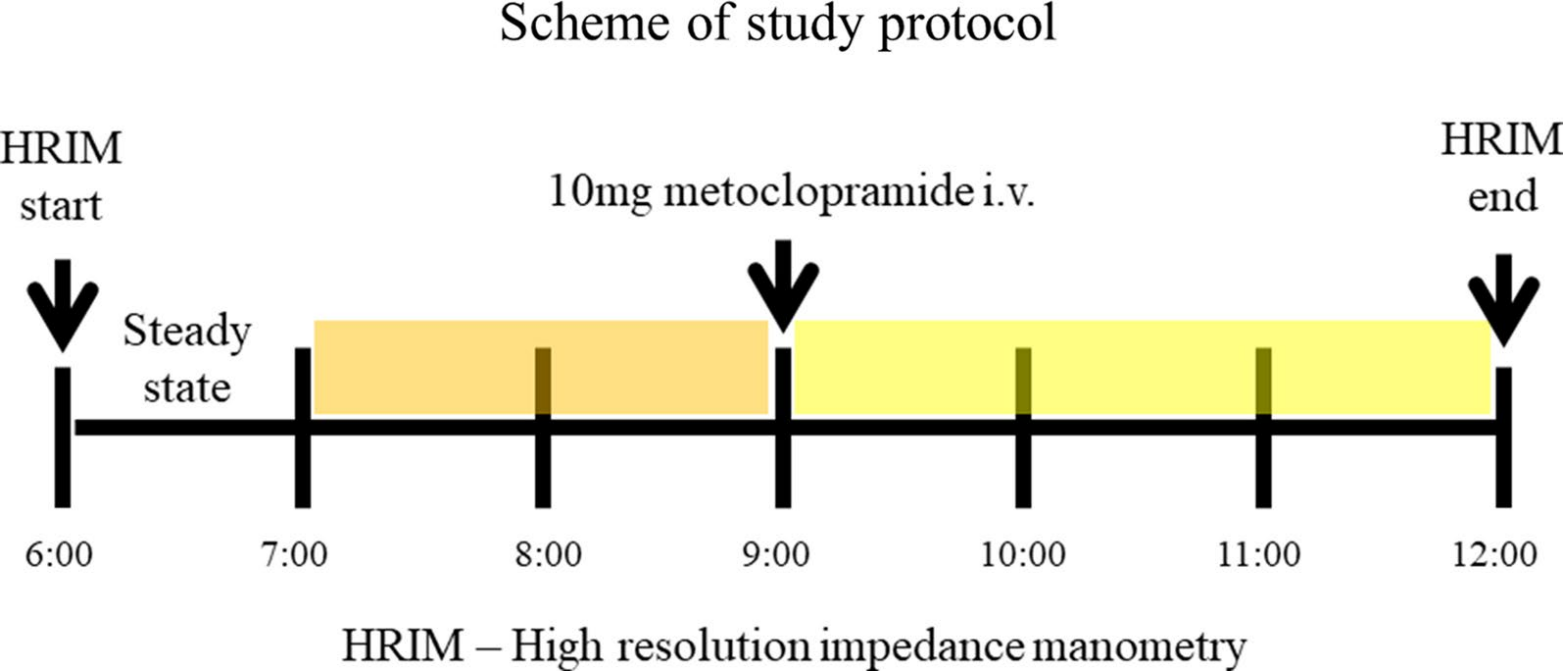
Scheme of study protocol

All critically ill patients received the same caliber of nasogastric tube (16 Fr) and the head of the bed was elevated to 30°. All patients received anti-ulcer prophylaxis using histamine 2-type receptor blockers or proton pump inhibitors, as well as a standard enteral nutrition formula (Nutrison Energy, 1.5 kcal/mL, Nutricia). Residual gastric volumes were assessed every 3 h via active suction. Sedation and/or analgesia were minimized in a goal-directed fashion and targeted light sedation (Richmond Agitation Sedation Score of − 2 to 0), with only propofol and/or sufentanil used to ensure the patient’s comfort. HRIM analysis included resting parameters of the esophagogastric junction (EGJ), dynamic parameters of all dry swallows, panesophageal secondary peristalsis, and analysis of reflux episodes according to the impedance measurements. A detailed description of the HRIM measurements for the critically ill patients is provided in Additional file 1. The study protocol scheme is shown in Fig. 1 and the monitored parameters are shown in Table 1.

**Table 1:** Variables monitored during HRIM study in critically ill patients

Clinical parameters:	Mean arterial pressure, heart rate, respiratory rate, oxygen saturation, body temperature (every hour), intraabdominal pressure (2 × during the study), ventilator setting
Laboratory parameters:	Arterial lactate, hemoglobin, creatinine, bilirubin, albumin, CRP, INR at 5:00, glycemia at 6:00, 9:00, 12:00
HRM resting parameters	Tonus of LES^a^ Inspiratory EGJ pressure (EGJP-insp). Average maximal inspiratory EGJ pressure for three respiratory cycles^a^ Expiratory EGJ pressure (EGJP-exp). Average EGJ pressure midway between inspirations for the same three respiratory cycles^a^ Esophagogastric junction contractile integral (EGJ-CI)^a^
HRM dynamic parameters	Number of dry swallows and panesophageal secondary peristalsis/1 h Distal contractile integral (DCI) % of failed peristalsis % of panesophageal pressurizations % of premature contractions % of double-peaked waves Integrated relaxation time (IRP) Intrabolus pressure (IBP)
Impedance	Number of liquid, mixed, and gaseous reflux episodes (defined as a sequential oral progressive decrease/increase in impedance below/above 40% baseline values distally with a retrograde propagation by at least 2 additional proximal observed fields in the absence of explanatory peristaltic component (median/hour of study) Number of distal and proximal refluxes Origin mechanism for each reflux episode: Swallow associated reflux (reflux within 10 s of finished swallow) Panesophageal secondary peristalsis associated reflux (reflux having TLESR character occurring within 10 s after panesophageal secondary peristalsis) Partial esophageal secondary peristalsis associated reflux (reflux having TLESR character occurring within 10 s after the non-swallowing partial esophageal activity) TLESR—reflux associated with a temporary decrease in LES tone below 5 mmHg, without association with dry swallow, esophageal secondary peristalsis or cough) Cough associated reflux (reflux within 30 s of cough episode/MV interference) Absence of the LES tone (reflux at low LES tone below 5 mmHg, without an association with esophageal contraction or cough) Panesophageal secondary peristalsis, partial esophageal secondary peristalsis in the close post-refulx period (within 10s of reflux) Cough Agitation

*HRM* high-resolution manometry, *LES* lower esophageal sphincter, *EGJ* esophagogastric junction, *TLESR* transient lower esophageal sphincter relaxation, *MV* mechanical ventilation

^a^Measured 3 times in each study hour and expressed as the median for a particular study period

### Statistical analysis

Data were presented as median (interquartile range, IQR) or number (percentage) unless otherwise indicated. Data from the first 2 h of the study were used to analyze the key features of esophageal motility functions in critically ill patients and to compare those features and the occurrence of reflux according to LGV or HGV status. The metoclopramide administration was started after the first 2 h of the study, and data from the following 3 h were used to perform a comparison of the parameters before and after the metoclopramide administration. The Mann–Whitney *U* test was used to compare two independent groups. Categorical data were compared by Fisher’s exact test. Differences were considered statistically significant at *p* values of < 0.05.

## Results

Demographic and clinical characteristics are presented in Table 2. The main diagnoses were septic shock (5 patients in the LGV and 7 patients in the HGV group), COPD exacerbation (2 patients in the LGV group), cardiogenic shock (1 patient in the LGV group), and aspiration pneumonia (1 patient in the HGV group). Major pathological motility patterns were observed in all patients (Table 3). Esophageal motility abnormalities included both disturbances of contraction vigor, contraction and pressurization patterns and EGJ functions.

**Table 2:** Demographic and clinical data of critically ill patients

Variable (unit)	Total *n* = 16	LGV group *n* = 8	HGV group *n* = 8
Age (years)	58 (51–71)	68 (52–76)	54 (51–64)
Body mass index	29 (25–34)	32 (24–33)	27 (25–35)
Sex—men (%)	44	13	75
Interval from admission to study (days)	5 (3–5)	4 (3–5)	5 (5–5)
Interval from MV start to study (days)	4.5 (3–5)	3.5 (3–5,3)	5 (4–5)
APACHE II score	35 (23–39)	35 (24–39)	33 (21–39)
SOFA score on the day of the study	11 (8–14)	10 (7–11)	14 (11–15)
Number of days in the ICU (days)	11 (14–19)	13 (11–19)	14 (12–18)
Duration of hospitalization (days)	26 (16–27)	24 (17–29)	26 (16–27)
ICU mortality (%)	25	25	25
Hospitalization mortality (%)	44	50	38
GRV 24 h before study (mL)	345 (28–630)	25 (8–68)	630 (627–985)
GRV single measurement during the study (mL)	30 (18–90)	15 (0–20)	90 (88–200)
Mean arterial pressure (mmHg)	92 (85–100)	94 (88–99)	90 (85–100)
Heart rate (beats/minute)	84 (74–104)	93 (79–103)	77 (71–100)
Intraabdominal pressure (mmHg)	10 (7–17)	15,5 (13–18)	7 (7–10)
Body temperature (°C)	37 (36–37)	37 (36–37)	36 (36–37)
Number of clinical interferences	1 (0–3)	2 (1–3)	1 (0–2)
Noradrenalin use during study (%)	31	25	38
Betablockers use during study (%)	6	13	0
Propofol use during study (%)	31	13	50
Sufentanil use during study (%)	56	38	75
Blood glucose (mmol/L)	10 (9–13)	10 (9–14)	11 (10–12)
Arterial lactate (mmol/L)	1,3 (0,8–1,7)	1,7 (1,4–2)	0,8 (0,8–1,3)
Hemoglobin (g/L)	102 (93–110)	104 (98–112)	98 (93–103)
C-reactive protein (mg/L)	72 (44–176)	68 (17–191)	72 (70–176)

All values shown are median and interquartile range or percentage

*LGV* low gastric volumes, *HGV* high gastric volumes, *MV* mechanical ventilation, *GRV* gastric residual volume, *ICU* intensive care unit, *LES* lower esophageal sphincter

**Table 3:** High-resolution manometry characteristics of critically ill patients

Variable (unit)	Critically ill patients *n* = 16	Normal range for 5 mL wet swallows (ref. 8 and 9)
**Dynamic (swallow) esophageal parameters**		
Distal contractile integral (mmHg cm s)	3852 (1700–7730)	> 450 and < 20% more than 8000
Failed esophageal peristalsis ( %, median/IQR)	73 (54–89)	< 20
Panesophageal pressurization (% median/IQR)	64 (48–79)	< 20
Double-peaked waves (% median/IQR)	30 (0–50)	≤ 15
Premature contraction (% median/IQR)	20 (2–32)	< 20
Rapid contraction (% median/IQR)	5 (0–12)	< 20
**Dynamic (swallow) EGJ parameters**		
Integrated relaxation pressure (mmHg, median/IQR)	16 (6–21)	< 15
Intrabolus pressure (mmHg, median/IQR)	23 (17–28)	< 17
**Static (rest) EGJ parameters**		
LES tone (mmHg, median/IQR)	18 (9–26)	13–43
EGJ-CI (mmHg cm, median/IQR)	40 (28–60)	25–55
Inspiration EGJ pressure (mmHg, median/IQR)	36 (29–42)	29–43
Expiration EGJ pressure (mmHg, median/IQR)	24 (19–33)	9–20
Hiatal hernia presence (%)	19	–

Normative data from healthy volunteers (ref. 8 and 9) are derived from measurements during water-swallowed induced esophageal contraction, whereas dry swallows or spontaneous contractions are analyzed in critically ill patients. Since quantitative differences in peristaltic variables exist between peristalsis associated with wet versus dry swallows, caution is needed when comparing them directly

*LES* lower esophageal sphincter, *EGJ* esophagogastric junction, *EGJ-CI *esophagogastric junction contractile integral

### Esophageal contraction vigor

The distal contractile integral (DCI) values, which reflect contraction vigor, were significantly higher in the patients than normally seen in healthy volunteers [8], which suggested that hypercontractile esophagus was common. In addition, 44% of the critically ill patients exhibited extreme contraction vigor, based on DCI values of > 8000 mmHg-cm-s for > 20% of esophageal events.

### Esophageal contraction pattern

Contraction pattern characterizes esophageal peristaltic integrity, i.e., if the peristaltic activity is intact, failed, or associated with peristaltic breaks. Despite the predominance of a hypercontractile pattern, simultaneously failed peristalsis (a propulsive contraction with a DCI < 100 mmHg-s-cm) was observed for 73% (IQR 54–89%) of the esophageal events in critically ill patients, which suggested an ineffective propulsive force. Significant irregularities in the contraction pattern were also observed, including double-peaked waves (30%, IQR 0–50%) and premature spastic contractions (20%, IQR 2–32%).

### Esophageal pressurization pattern

Major abnormalities were detected in the pressurization patterns, with the most common being panesophageal pressurization (64%, IQR 48–79%). This pattern involves impaired integrity of the contraction (achalasia type II phenotype), which includes pressurization of the entire esophagus from the upper sphincter to the esophagogastric junction (EGJ).

### EGJ functions

**Fig. 2:**
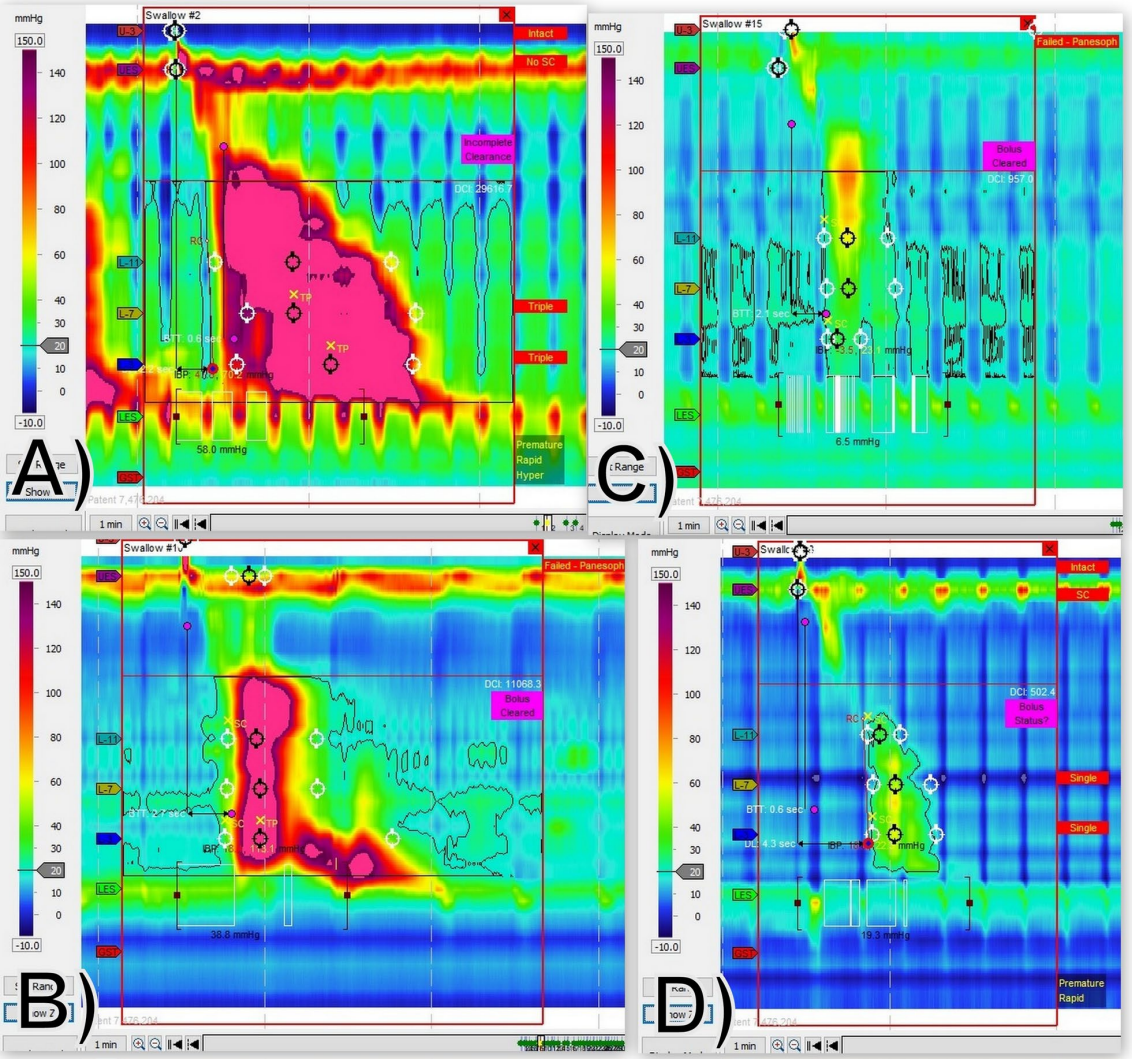
Most common esophageal motor pattern in ventilated critically ill patients. **a** Swallow followed by hypercontractile, spastic contraction, and poor LES relaxation (distal contractile integral 29,617 mmHg cm s, distal latency 2.2 s, integrated relaxation pressure of LES 58 mmHg). **b** Swallow followed by hypercontractile panesophageal pressurization and poor LES relaxation (distal contractile integral 11,068 mmHg cm s, integrated relaxation pressure of LES 39 mmHg). **c** Swallow followed by normocontractile panesophageal pressurization and normal LES relaxation (distal contractile integral 957 mmHg cm s, integrated relaxation pressure of LES 6.5 mmHg). **d** Swallow followed by spastic contraction with normal contraction vigor and poor LES relaxation (distal contractile integral 502 mmHg cm s, distal latency 4.3 s, integrated relaxation pressure of LES 19 mmHg). *LES* lower esophageal sphincter

Several dynamic and static parameters were evaluated to characterize EGJ function (Table 1). Among these parameters, 50% of patients had an increased integrated relaxation pressure (> 15 mmHg) and 63% of patients had an intrabolus pressure of > 20 mmHg, which both reflect EGJ outflow obstruction during esophageal contraction. A novel static parameter for evaluating the rest LES barrier function (the EGJ-CI parameter) [9] was decreased (< 13 mmHg cm) in only 1 patient (hypocontractile type) and was increased (> 55 mmHg cm) in 31% of patients. Representative examples of the HRIM findings are shown in Fig. 2.

### Comparison of LGV and HGV groups

When we compared the manometric parameters according to the feeding tolerance, the LGV group commonly had the hypercontractile pattern with panesophageal pressurization, while the HGV group had the normocontractile pattern with failed peristalsis (Table 4, Additional file 2). Relative to the HGV group, the LGV group generally had more esophageal events, which often involved premature peristalses and rapid contractions, and also had more double-peaked waves. Moreover, the LES had a higher tone in the LGV group and significantly higher integrated relaxation pressure, which suggested EGJ outflow obstruction. In contrast, only patients in the HGV group (31%) had a LES with a lower tone (< 13 mmHg).

**Table 4:** Characteristics of HRIM parameters according to feeding tolerance

Variable (unit)	LGV group *n* = 8	HGV group *n* = 8
Number of panesophageal events /h	17 (11–26)	6 (4–8)*
Distal contractile integral (mmHg cm s, median/IQR)	7661 (1580–11,908)	3111 (1700–5068)
Failed esophageal peristalsis (%, median/IQR)	62 (50–73)	85 (69–98)
Panesophageal pressurization (%, median/IQR)	62 (50–73)	69 (43–83)
Double-peaked waves (%, median/IQR)	48 (43–51)	0 (0–5)*
Premature contraction (%,median/IQR)	31 (25–37)	2 (1–19)*
Rapid contraction (%, median/IQR)	12 (11–22)	0 (0–0)*
Integrated relaxation pressure (mmHg, median/IQR)	20 (16–28)	6 (6–16)
Intrabolus pressure (mmHg, median/IQR)	19 (16–27)	25 (19–30)
LES tone (mmHg, median/IQR)	25 (19–31)	9 (8–16)*
EGJ-CI (mmHg cm, median/IQR)	53 (40–60)	30 (20–50)
Inspiration EGJ pressure (mmHg, median/IQR)	40 (35–48)	34 (28–36)
Expiration EGJ pressure (mmHg, median/IQR)	30 (22–35)	20 (19–25)
Hiatal hernia presence (%)	13	25

*LGV* low gastric volumes, *HGV* high gastric volumes, *LES* lower esophageal sphincter, *EGJ-CI* esophagogastric junction contractile integral

*LGV versus HGV, Man Whitney test, *p* < 0.05

### Reflux testing in critically ill patients

**Fig. 3:**
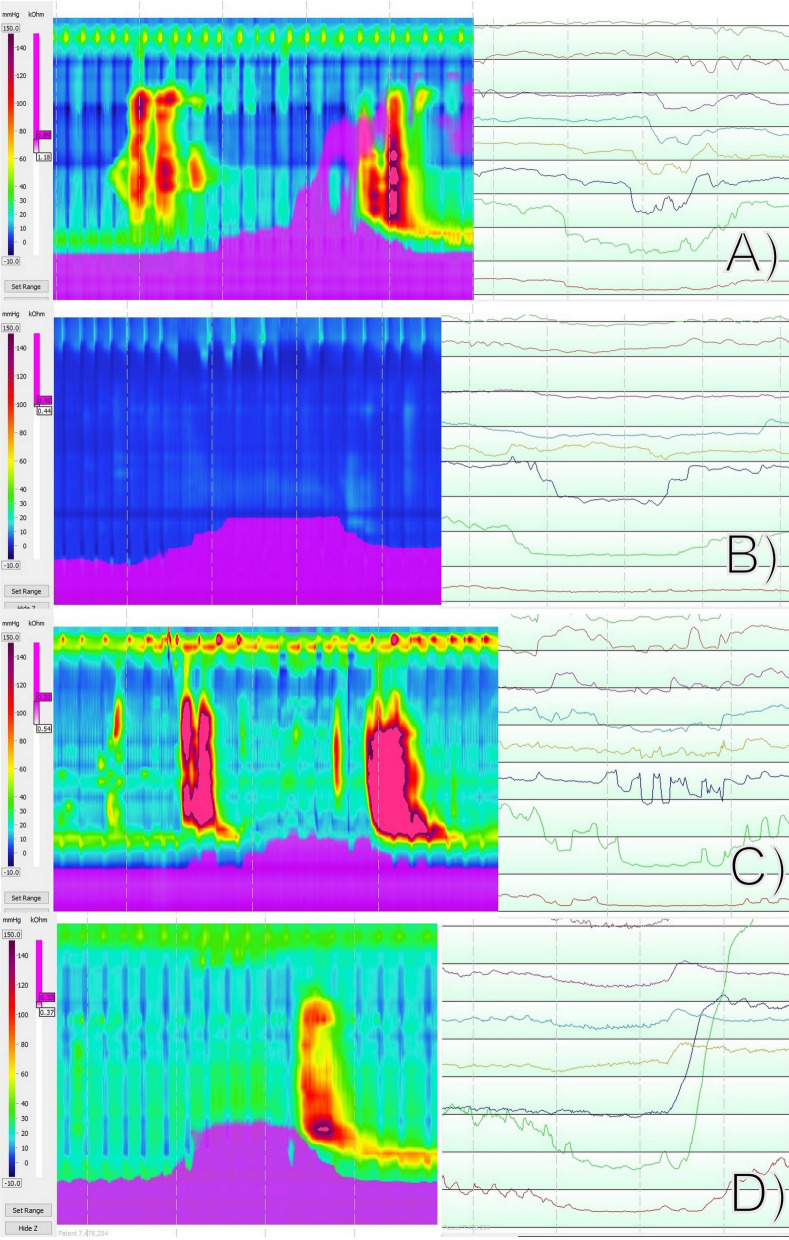
High-resolution impedance manometry picture of most frequent reflux types in critically ill patients. On the right high-resolution impedance manometry flowchart, on the left impedance curves for the same time period. **a** proximal reflux associated with transient lower esophageal sphincter relaxation (TLESR) with preceding panesophageal secondary peristalsis and terminated by secondary peristalsis. **b** Distal reflux in the absence of lower esophageal sphincter tone. **c** Distal reflux associated with dry swallow and terminated by secondary peristalsis. **d** Distal reflux associated with TLESR without preceding secondary peristalsis terminated by secondary peristalsis

Table 5 shows the impedance testing results. During the 5-h study period, a total of 195 reflux episodes were observed among all patients (11; IQR: 4–18) and reflux prevalence was 81% during the first 2 h of the study (i.e., at least 1 reflux episode before the metoclopramide administration). There was substantial heterogeneity as the number per patient ranged between 0 and 34 (Additional file 3). Thirty-eight episodes (19.5%) were proximal (i.e., reflux reaching the border of the upper esophageal sphincter), with most being mixed (19 episodes, 50%) or liquid (17 episodes, 45%) and only 2 episodes being gaseous. Reflux was most commonly associated with transient lower esophageal sphincter relaxation (TLESR, defined as LES relaxations in the absence of swallow; 133 out of 195 episodes, 68%). In addition, TLESR was preceded by panesophageal secondary peristalsis in 90 of all reflux episodes (46%). By contrast, reflux associated with absence of LES tone was less common (35 episodes, 18%). Similarly, 12 episodes (6%) were associated with cough, ventilator interference, or agitation. Representative examples of the most common reflux types based on the HRIM images are shown in Fig. 3. Interestingly, the total number of reflux episodes was noticeably higher in the LGV group than in the HGV group (Table 5). Moreover, two different esophageal motility patterns were associated with the reflux, as most episodes in the LGV group (76/121 episodes, 63%) were associated with TLESR preceded by panesophageal secondary peristalsis, while episodes were commonly associated with absence of LES tone in the HGV group (34/74 episodes, 46%).

**Table 5:** Impedance analysis of critically ill patients in the LGV and HGV group

Reflux type (number of all refluxes in the group)	Total *n* = 16	LGV group *n* = 8	HGV group *n* = 8
Swallow (primary peristalsis) associated reflux	21	16	5
Reflux in TLESR with preceding panesophageal secondary peristalsis	90	76	14
Reflux in TLESR with preceding partial esophageal secondary peristalsis	25	12	13
Reflux in TLESR without preceding esophageal secondary peristalsis	18	11	7
Absence of the LES tone	35	1	34
Cough associated reflux	6	5	1
Total	195	121	74

*LGV* low gastric volumes, *HGV* high gastric volumes, *LES* lower esophageal sphincter, *TLESR* transient lower esophageal sphincter relaxation

### Effects of metoclopramide

In critically ill patients (Table 6), metoclopramide significantly decreased the total number of esophageal events, especially in the LGV group, but did not influence panesophageal pressurization or premature contraction. Similarly, there was a tendency toward reduced contraction vigor, based on the DCI value, which was predominantly observed in the LGV group. Nevertheless, metoclopramide appeared to exert different effects on the EGJ function and LES barrier function in the two groups. In the LGV group, metoclopramide significantly improved LES relaxation during esophageal activity (based on a reduced integrated relaxation pressure value), but impaired the resting LES barrier function (based on a decreased EGJ-CI value). In contrast, metoclopramide in the HGV group improved the vigor of the distal esophageal contraction (based on inspiration and expiration EGJ pressures) and thereby improved the EGJ barrier function. Nevertheless, metoclopramide failed to change the number reflux episodes in either group.

**Table 6:** HRIM analysis before and after metoclopramide administration in critically ill patients

Variable (unit)	Before metoclopramide			After metoclopramide		
	Total	LGV	HGV	Total	LGV	HGV
GRV during the study (mL)	30 (18–90)	15 (0–20)	90 (88–200)	15 (10–160)	10 (0–10)	160 (88–213)
Number of esophageal activities /hour	10 (6–17)	17 (11–26)	6 (4–8)	7 (5–13)*	11 (7–15)*	5 (4–9)
Distal contractile integral (mmHg cm s)	3852 (1700–7730)	7661 (1580–11,908)	3111 (1700–5068)	3004 (1900–8091)	5096 (1699–8916)	2805 (2081–4488)
Failed esophageal peristalsis (median of %)	73 (54–89)	62 (50–73)	85 (69–98)	80 (58–100)	68 (53–85)	94 (69–100)
Panesophageal pressurization (median of %)	64 (48–79)	62 (50–73)	69 (43–83)	74 (45–88)	68 (53–85)	80 (45–90)
Double-peaked waves (median of %)	30 (0–50)	48 (43–51)	0 (0–5)	0 (0–25)**	25 (8–35)*	0 (0–0)
Premature contraction (median of %)	20 (2–32)	31 (25–37)	2 (1–19)	10 (0–41)	33 (10–48)	0 (0–3)
Integrated relaxation pressure (mmHg)	16 (6–21)	20 (16–28)	6 (6–16)	14 (7–19)	17 (14–24)*	7 (6–10)
Intrabolus pressure (mmHg)	23 (17–28)	19 (16–27)	25 (19–30)	24 (20–27)	24 (21–26)	24 (19–29)
LES tone (mmHg)	18 (9–26)	25 (19–31)	9 (8–16)	18 (12–27)	23 (18–30)	12 (9–20)
EGJ-CI (mmHg cm)	40 (28–60)	53 (40–60)	30 (20–50)	33 (2–53)	43 (30–53)*	30 (26–44)
Inspiration EGJ pressure (mmHg)	36 (29–42)	40 (35–48)	34 (28–36)	42 (30–44)	40 (34–45)	42 (27–44)*
Expiration EGJ pressure (mmHg)	24 (19–33)	30 (22–35)	20 (19–25)	25 (23–32)	27 (24–32)	23 (21–29)*
Number of reflux episodes/patient/hour	1.8 (0.5–3.5)	3.3 (2–3.6)	0.8 (0.4–1.5)	2.4 (0.9–3.6)	2.9 (2.3–3.7)	1.1 (0.5–2.6)

All values shown are median and interquartile range

*GRV* gastric residual volume, *LES* lower esophageal sphincter, *EGJ-CI* esophagogastric junction contractile integral

*The Mann–Whitney U test, p < 0,05

## Discussion

This is the first study to use HRIM to characterize esophageal motor activity, reflux mechanisms, and response to metoclopramide in mechanically ventilated critically ill patients during their early recovery phase. The most common patterns were hypercontractility with premature contractions, ineffective propulsive force, and panesophageal pressurization. Gastroesophageal reflux predominantly occurred due to TLESR that was often preceded by panesophageal secondary peristalsis, and reflux was only associated with the absence of LES tone and episodes of straining in a minority of patients. In addition, although all patients had major abnormalities in their contraction vigor and contraction patterns, their characteristics varied according to gastric nutritional tolerance, which is a surrogate parameter for gastrointestinal dysfunction. For example, the LGV group was more likely to have the hypercontractile pattern with panesophageal pressurization, EGJ outflow obstruction, and more frequent reflux. In contrast, delayed gastric emptying was associated with the normal contraction vigor, failed peristalsis, reduced LES tone, and paradoxically fewer reflux episodes. In addition, metoclopramide had opposite effects on the EGJ barrier function in the LGV and HGV groups, but failed to alter the number of reflux episodes in either group.

Very few studies have evaluated quantitative and qualitative physiological parameters of esophageal motility in critically ill [2, 3, 5], and none of those studies have used HRIM in mechanically ventilated patients. The introduction of HRIM in the field of gastroenterology led to the development of novel objective metrics and patterns for describing esophageal motility functions [9]. Hypercontractility with panesophageal pressurization was the most common motor abnormality in our patients, although we observed a very complex spectrum of altered esophageal physiologies. In this context, our study is markedly different from the study performed by Kölbel et al., who used conventional manometry and reported that considerable esophageal hyporeactivity was characterized by reduced propulsive motility, in terms of both the frequency and amplitude of contractions [2]. The difference between their findings and ours might be related to the more intensive analgosedation protocol that they used. Our findings also provide a strong physiological explanation for the negative results of a recent study, which revealed that esophageal electrical stimulation failed to prevent regurgitation and enhance feeding [10].

Another major feature of this study is the evaluation of esophageal physiology in two different gastric motility states. We observed that the LGV group had significantly more esophageal activities than the HGV group, which were associated with greater contraction vigor, more double-peaked waves, and premature and rapid contractions. These factors all reflect spastic peristalsis. Interestingly, a substantial proportion of patients exhibited extremely vigorous peristalsis, which is a pattern that resembles jackhammer (hypercontractile) esophagus [8]. The hyperreactive pattern that was more common in the LGV group was also associated with signs of EGJ outflow obstruction, while ineffective esophageal motility with reduced LES tone was only observed in the HGV group. These intergroup differences may have several explanations. First, the HGV group tended to have higher SOFA scores on the day of measurement and more severe acute kidney injury, which both suggests more severe illness. Furthermore, more frequent use of analgosedation could negatively affect gastrointestinal motility and enteral feeding tolerance in the HGV group [11], whereas the less frequent use of propofol and sufentanil could make the LGV patients more sensitive to irritation caused by the endotracheal and/or nasogastric tubes. Nevertheless, that reasoning is speculative, as the analgosedation was titrated to target the same Richmond Agitation Sedation Score in both groups. Our results may also reflect a physiological connection between esophageal and gastric motility patterns. Indeed, the greater motility in the UGIT of patients in the LGV group might be associated with very good gastric feeding tolerance. However, it was also accompanied by esophageal hypercontractility and EGJ outflow obstruction. The clinical significance of this pattern remains unclear and is it not necessarily indicative of an underlying pathology.

Abnormal esophagus motility disorders in mechanically ventilated patients could be associated with significant clinical consequences, which are related to gastroesophageal reflux and pulmonary aspiration [1]. In this context, low LES pressure and weak or even absent esophageal peristalsis has been traditionally considered crucial for the development of gastroesophageal reflux in critically ill patients [2]. For example, Nind et al. reported that low or absent LES pressure and inadequate esophageal propulsive forces led to poor acid clearance, which was considered the most common mechanism associated with reflux [3]. However, our data suggest that maintaining rest LES tone may not be essential for preventing reflux, as almost 70% of the reflux episodes we observed occurred due to TLESR. Although TLESR may be a normal physiological motor event in healthy subjects [12], previous studies have proven that TLESR plays a crucial role in the occurrence of gastroesophageal reflux disease [13]. It appears that the major mechanisms of reflux in our cohort of patients recovering from critical illness and receiving minimal sedation are similar to non-ventilated patients. By contrast, a notable feature of esophageal activity that often preceded TLESR was panesophageal secondary peristalsis, an abnormality never seen in healthy subjects [12]. It remains unclear, whether this secondary peristalsis is itself a trigger of TLESR. Of note, the absence of LES tone was associated with only a small minority of reflux episodes (18%), and this situation was only observed in patients with HGV. Furthermore, high residual gastric volumes are commonly thought to increase the risk of gastroesophageal reflux [14]. Interestingly, patients in the LGV group had substantially better gastric emptying but also more reflux episodes than patients in the HGV group. Our observations are corroborated by the findings reported by McClave et al., who were unable to identify a threshold for residual gastric volumes that was associated with increased risks of aspiration and pneumonia [15]. Thus, it appears that reflux disease cannot be excluded based on the presence of well-preserved gastric emptying, maintained LES tone, and well-preserved EGJ barrier function in critically ill patients.

Metoclopramide is frequently used to promote motility in the UGIT. However, its beneficial effects remain unclear in critically ill patients [16], and we are not aware of any comprehensive studies regarding the effects of metoclopramide on esophageal physiology and EGJ function. We observed that metoclopramide did not substantially reduce the number of reflux episodes in either group of critically ill patients. In addition, its effects on esophageal functions are noticeably different in the healthy volunteers, where it markedly increases the contraction vigor [15]. Across the observed spectrum of abnormal motility patterns, metoclopramide also paradoxically decreased the number of esophageal events and even tended to weaken the strength of contractions, especially in the LGV group. The mechanisms underlying these unexpected effects remain unclear. In addition, metoclopramide did not substantially influence failed esophageal peristalsis, esophageal pressurization, or EGJ-related parameters. Thus, HRIM does not appear to be able to clinically identify critically ill patients who are likely to benefit from prokinetic stimulation.

This study has several limitations. First, the small sample size may preclude generalization of the results because of selection bias, although this study was only designed as an exploratory physiological study. Focusing on population with “normal” and “delayed” gastric emptying, we studied only patients with two pre-defined, well-separated thresholds for gastric residuals. Hence, the results might not be valid for a wider spectrum of gastric feeding tolerance. Likewise, the observed relationships between reflux and different esophageal patterns should be interpreted with caution as they may be influenced by a correlation within a small number of patients. Second, critical illness is a dynamic process and analyses from a single timepoint only provide a limited perspective regarding these patients. Thus, we suggest that further studies are needed to assess the dynamic long-term implications of our HRIM findings. Third, the definition of abnormalities described in the Chicago classification is based on the analysis of ten 5-ml swallows in patients suffering from dysphagia and/or chest pain and therefore may not necessarily apply to the manometric analysis in ICU patients, in whom dry swallows or spontaneous secondary peristalsis were studied. Finally, all our critically ill patients received anti-ulcer prophylaxis and pH monitoring was not performed for various reasons, including the technical complexity of introducing a pH-meter catheter to the nasogastric tube and HRIM catheter.

## Conclusions

In conclusion, this study revealed that HRIM was useful for clarifying the complex esophageal physiology in critically ill patients. HRIM testing in critically ill patients has been found safe and feasible. The results revealed marked heterogeneity in the esophageal motor dysfunctions, as well as links between gastric emptying, esophageal motor patterns, and gastroesophageal reflux. Our study also provides valuable insights into the physiological effects of metoclopramide in this population. Nevertheless, additional studies are needed to better understand the clinical relevance and implications of our findings.

## Supplementary information

**Additional file 1**. Study protocol and high-resolution impedance manometry record analysis details.

**Additional file 2**. High-resolution motility data in critically ill patients.

**Additional file 3**. Number of reflux episodes for the entire of recording period.

## Data Availability

The data that support the findings of this study will be available from the first author upon reasonable request (email: balihar@fnplzen.cz). Supporting data will be made available to Editorial Board Members and referees at the time of submission for the purposes of evaluating the manuscript and directly upon request to any reader on and after the publication date. Supporting datasets will be made available as Supplementary Information files that will be freely accessible on the journal’s website upon publication.

## Abbreviations

DCI: Distal contractile integral
EGJ: Esophagogastric junction
GRV: Gastric residual volume
HGV: High residual gastric volumes
HRIM: High-resolution impedance manometry
LES: Lower esophageal sphincter
LGV: Low residual gastric volumes
TLESR: Transient lower esophageal sphincter relaxation
UGIT: Upper gastrointestinal tract

## Code availability

License for High resolution manometry software: ManoScan ESO: NLJIHFEDCA, ManoView ESO: 21-E413-9824.
