# Supplementary material for: Characterization of esophageal motor activity, gastroesophageal reflux, and evaluation of prokinetic effectiveness in mechanically ventilated critically ill patients: a high-resolution impedance manometry study
Source: Crit Care. 2021 Feb 8;25:54. doi: 10.1186/s13054-021-03479-8 (PMC7870125; doi:10.1186/s13054-021-03479-8)
Supplement: Supplementary file 1 — Additional file 1. Study protocol and high-resolution impedance manometry record analysis details. [file 13054_2021_3479_MOESM1_ESM.docx]

**Additional file 1**

**Title:** Study protocol and high-resolution impedance manometry record analysis details

**Study protocol**

**Protocol for HGV a LGV group:**

The study protocol for critically ill patients is schematically shown in Figure 1. Non-consecutive patients were selected after admission to the ICU, and if the inclusion criteria were met, treatment was standardized and relatives were contacted to provide informed consent. If the total residual gastric volume met inclusion criteria for either the LGV or HGV group (evaluated always at 5:00am on the day of the expected enrollment in the study), the patient was enrolled and the HRIM catheter (ManoScan TM System, Given Imaging, HRM catheter with impedance ref. MSC-3890-Z) was calibrated between 5:30 a.m. and 6:00 a.m. Pressure calibration and thermal calibration in a water bath at 37 deg. Celsius were performed. When night sedation was still in effect the calibrated catheter lubricated with a gel with local anesthetic was inserted along the nasogastric tube (NGT) into the stomach so that the upper and lower esophageal sphincter and stomach pressure were visible in the recording window and monitoring of esophageal pressure and impedance was started. Night sedation was terminated or minimized at 6:00 a.m. which was followed by a 1-hour period to reach a steady state. From 7:00 a.m. onwards, HRIM study data were collected and other clinical and laboratory parameters were monitored for 5 hours while on continuous enteral nutrition (see Online resource 3 for definitions). At 9:00 a.m., all patients received a single dose of metoclopramide 10 mg i.v. and monitoring continued until 12:00 a.m. Throughout the study, instructed experienced nurse recorded the time of oral and tracheal suction, cough or agitation, necessary manipulation and bolus drug delivery to NGT. NGT residues were checked at 7:30 a.m. and 10:30 a.m. Intra-abdominal pressure was measured at 6:30 a.m. and at 11:30 a.m. Potentially interfering co-medication (beta-mimetics, beta-blockers, parasympatholytics, etc.) was also recorded. At 12:00 a.m., the study was terminated and the catheter was extracted.

**Exact HRIM data analysis**

All HRIM records were analysed using The ManoView^TM^ ESO Analysis Program Version 3.

**HRIM record analysis in critically ill patients:**

After opening each record, a two-point thermal calibration was first performed (when HRIM catheter is in the water bath before introducing it into the patient and just after withdrawing the catheter from the patient). Subsequently, the recording time was synchronized with the study time to identify the beginning of each study hour. Then, within each 1-hour period of recording, resting HRM parameters were measured in 3 periods (10.-15., 25.-35. and 45.-55. minute) so that the landmark window was placed ideally in a quiescent portion of recording, free of swallows. Thus, a total of 15 values ​​for each parameter during the 5-hour study were recorded. Subsequently, a landmark period in the 25.-35. minute was evaluated for each study hour separately, identifying all panesophageal events (dry swallows and panesophageal secondary peristalsis) [1], setting all swallow windows and manually revising / adjusting bars in each window separately to obtain dynamic parameter values ​​for all individual esophageal event (Additional file 2). IRP was also measured for panesophageal secondary peristalsis that has no prior swallowing.

All reflux episodes were identified in the impedance recording, their characteristics and cause were determined (Table 5). The changes in impedance accompanying esophageal contraction were not recorded as reflux. According to the nurse's records, data on when the patient was agitated, vomited, received medication, coughed, etc. were added and correlation with these symptoms or actions was determined.

All records were first evaluated by one physician skilled in HRIM functional diagnostics and then independently critically assessed by a second equally experienced physician from the author team (especially location of landmark windows, identification of panesophageal events and manual analysis of individual swallows and secondary peristalses - bar settings, reflux identification and evaluation of their cause). Possible disagreements were resolved by joint decision after discussion.

Reference:

1 He S, Jell A, Hüser N, Kohn N, Feussner H. [24-hour monitoring of transient lower esophageal sphincter relaxation events by long-term high-resolution impedance manometry in normal volunteers: The "mirror phenomenon".](https://pubmed.ncbi.nlm.nih.gov/30676686/) Neurogastroenterol Motil. 2019; doi: 10.1111/nmo.13530.
