# Supplementary material for: Characterization of esophageal motor activity, gastroesophageal reflux, and evaluation of prokinetic effectiveness in mechanically ventilated critically ill patients: a high-resolution impedance manometry study
Source: Crit Care. 2021 Feb 8;25:54. doi: 10.1186/s13054-021-03479-8 (PMC7870125; doi:10.1186/s13054-021-03479-8)
Supplement: Supplementary file 2 — Additional file 2. High-resolution motility data in critically ill patients. [file 13054_2021_3479_MOESM2_ESM.pdf]

Additional file 2

Title: High resolution manometry data in critically ill patients

Patient number	Study group	DCI (mmHg.s.cm)	Failed peristalsis (%)	Panesophageal pressurisation (%)	Premature contraction (%)	IRP (mmHg)	IBP (mmHg)	Tone LES (mmHg)	EGJ-CI (mmHg.cm)	Insp. EGJ pressure (mmHg)	Exp. EGJ pressure (mmHg)
1	LGV	9 837	37	37	78	17,6	21,7	16,6	64,5	27,5	32,5
2	LGV	15 322	59	59	28	21,8	14,3	29,4	38	48,5	39
3	LGV	24 914	70	70	30	51,15	40,85	59,65	168,5	110,5	61,5
4	LGV	1 838	54	54	46	13,4	26,2	14,4	24	39,5	22,5
5	LGV	10 770	88	88	11	37,1	16,6	36,1	52,5	48	34
6	LGV	5 485	82	82	18	10,15	27,75	25,6	53	39,5	28
7	LGV	804	66	66	34	25,4	16,6	24,2	59	24	13,5
8	LGV	502	27	25	32	18,7	15,35	19,75	40,5	37	22
9	HGV	4 594	75	75	0	6,05	20,45	7,9	22,5	40	18,5
10	HGV	1 757	79	79	22	20,1	16,4	21,1	39,5	29	19
11	HGV	7 028	100	100	0	19,15	29,8	14,4	82,5	34,5	28
12	HGV	3 111	52	52	34	5,9	31,6	6,4	29,5	26	19,5
13	HGV	1 526	97	97	3	3,4	24,75	8,75	13,5	33,5	24,5
14	HGV	3 111	91	63	0	6,05	24,75	8,75	29,5	33,5	20,5
15	HGV	6 493	18	18	18	14,7	40,9	26	90	48,5	36,5
16	HGV	4	100	0	0	5,7	4,6	7,9	0,75	8	3,5

DCI - distal contractile integral; IRP - integrated relaxation pressure; IBP - intrabolus pressure; LES - lower esophageal sphincter; EGJ-CI - esophagogastric junction contractile integral; EGJ - esophagogastric junction; LGV - low residual gastric volumes; HGV - high residual gastric volumes
