# Supplementary material for: Characterization of esophageal motor activity, gastroesophageal reflux, and evaluation of prokinetic effectiveness in mechanically ventilated critically ill patients: a high-resolution impedance manometry study
Source: Crit Care. 2021 Feb 8;25:54. doi: 10.1186/s13054-021-03479-8 (PMC7870125; doi:10.1186/s13054-021-03479-8)
Supplement: Supplementary file 3 — Additional file 3. Number of reflux episodes for the entire of recording period. [file 13054_2021_3479_MOESM3_ESM.pdf]

Additional file 3

Title: Number of reflux episodes for the entire of recording period

Patient number	Study group	Number of refluxes/study period
1	LGV	13
2	LGV	33
3	LGV	2
4	LGV	4
5	LGV	22
6	LGV	16
7	LGV	17
8	LGV	14
9	HGV	34
10	HGV	5
11	HGV	3
12	HGV	19
13	HGV	0
14	HGV	8
15	HGV	5
16	HGV	0
**Total**	**---**	**195**

LGV - low gastric volumes; HGV - high gastric volumes;
